# Obstetric Anæsthesia

**Published:** 1888-03

**Authors:** N. M. Dodson

**Affiliations:** Berlin, Wis.


### OBSTETRIC ANÆSTHESIA.

BY DR. N. M. DODSON, BERLIN, WIS.

January 19, 1847, Sir Jas. T. Simpson first tried anæsthesia with ether in a midwifery case, in which a deformed pelvis necessitated version. The following day he triumphantly announced his complete success to the Edinburgh Obstetrical Society. November, 1847, he substituted chloroform for ether with the same happy result. The civilized world hailed his discovery with delight, and his numerous followers have shown accumulated abundant proof of the validity of his positicn. Yet, despite the successful use of anæsthetics in thousands of labor cases during the past four decades, their use is clearly the exception rather than the rule. The belief seems to obtain both with the profession and public that only in special and exceptional cases is anæsthesia indicated. Yet nothing in the experience or teachings of the great obstetricians can be found to account for this belief.

Dr. Simpson regarded anæsthetics in labor as perfectly safe, and experience subsequent to his untimely death has but corroborated his views. Charpentier, Gillette, Vergeley, Barker, Parvin, Lusk and the majority of obstetricians strongly commend the use of aæsthetics in natural labor. Parvin states that the only case of death ever reported as due to anæsthetics in labor lacked post-mortem confirmation. Fordyce Barker has seen but one case of post-partum hemorrhage occur among the thousands of labor cases in which he used anæsthetics. Our own J. K. Bartlett has recently stated that if post-partum hæmorrhage ever occur as a result of anæsthesia, the anæsthetic employed is impure or has not been used with due discretion. Dr. Claibourne concludes, after a careful analysis of the literature and of his own experience ; First, that the process of labor may be facilitated in all its stages by the use of chloroform ; second, that the duration of labor in all its stages may be shortened by its use ; third, that by it the pains of labor may be entirely obtunded with perfect safety ; fourth, that the accidents of labor in all its stages occur less frequently under the use of chloroform.

Despite the suffering which comes under their notice, physicians probably still fully appreciate the agony and terror of the fearful ordeal some women must endure whenever a human being is brought into the world. Simpson says that custom, prejudice and the idea that it is inevitable, lead the profession and their patients to look upon the amount and intensity of pain endured in ordinary labor as unworthy of very serious consideration. The actual pain endured in an average labor is far greater than that attendant upon most major surgical operati ns. Merriam, referring particularly to the passage of the child's head over the perineum, describes it as almost beyond human endurance. Velpeau speaks in very emphatic terms of "those piercing cries, that so lively agitation, those excessive efforts, those inexpressible agonies and those seemingly intolerable pains which accompany parturition's termination in woman." All this each one of us has observed time and again. Crediting ourselves with full sympathy with the delicate creature suffering the untold agony before us, we have withheld the "lethean" which would have made the agony endurable or turned into sweet oblivion with increased certainty of recovery. To her pleading cries the only answer has been, "Oh, be patient. Nature must take its course. You're doing well." If the agonies of natural labor be as described, what shall be said of complicated labor, with a contracted pelvis, a disproportionate head, a slowly dilating os, a rigid perineum, a faulty presentation necessitating version or more serious opperation? All the instruments of torture devised by mediæval tyrants failed of the agony which labor entails upon woman's delicate body. The sufferings of the "Son of Man" command the pity and adoration of the civilized world, yet who shall say that greater agony was not endured by the patient mother who bore Him in the stable of Bethlehem, "when there was no room in the inn."

The fact that in all ages the agony of childbirth has been borne without relief by unaccounted millions of mothers, does not absolve us from our great duty of relieving pain when we can do so easily and safely. What surgical operation compares in its powers of producing pain to childbirth, yet who would dare even to evulse an ingrowing nail without anæsthesia?

The relation of anæsthetics to labor need not here be discussed in regard to their kinds, doses and modes of application. It is sufficient to say that by them labor may safely be made endurable and shorn of its terrors which so appall womankind.

We, perhaps, little appreciate the dread with which this event is associated in the minds of our mothers, sisters, wives and daughters, nor how much it has to do with the crying sin of our age —fœticide. With the advance of civilization the increased education and enlightenment of mankind, the increasing delicacy of nervous organization with resultant increased susceptibility to pain, with the knowledge that somehow childbearing can be avoided, have brought into existence the professional abortionist. Fœticide is committed occasionally, when a pitying eye would weep for the poor deluded girl, who, more sinned against than sinning, would hide her degradation at the risk of her life. But here is not the harvest of the wretch who lives that the unborn may die. He reaps it from the woman who would glory in maternity but for its terrors, and who submits to the degradation that she may escape the rack of childbirth.

This knowledge seems almost intuitive. The maiden goes to the bridal bed all love and confidence, but whispers, "How shall we avoid children?" Then follow months of doubt, fear, and terror untold, and, finally when the ordeal is over, the resolution not to again undergo its agonies is universal. Who of us is not besieged with demands for the means to prevent conception or destroy its product? We urge the danger to life, the almost certain permanent injury to health, the destruction of family, happiness and the awful stigma of crime, but are met with the answer: "I know it is wrong—it is a crime; I know the danger to life and health; no matter. I'll give you all you ask; do anything you demand, but I'll never again endure the agony of childbirth, if I can avoid it." And so, in spite of your advice, she, on your refusal to aid, turns to the abortionist and sacrifices, sometimes, her health, always a large fee, and the price, we have every reason to believe, usually includes her honor, which in her desperation is forfeited despite all her pride and delicacy.

This is no fancy sketch. The fact that the best and most cultivated people of the land raise on an average less than one child each, and that the rising generation consists of by far too large a proportion of children of the imported lower classes, tells us in no uncertain tones that there is much truth here. We may pride ourselves that our skirts are clean, but is it true that we are wholly innocent of responsibility in this matter? Is not most of this crime the result of the dread of the ordeal of childbirth? What woman grudges the care of her living child or would part with it, aye, would not die for it, if necessary? What woman looks forward with any unwillingness to give up the pleasures of society for her offspring? If we can say to every woman who dares approach us with the proposition of fœticide: "Go home, take the best of care of your health; give up all dread of the event you are approaching, and I will see the period of your accouchment only a blissful memory or a day not at all to be dreaded. You shall sleep when nature exacts most of you and you shall be abundantly rewarded for the little pain you suffer;" can we not thus bless the race? Can we not be true to every instinct of humanity and gallantry, and so hold the honored place of family physician that no man may come between us and the women of our clientelle in that entire and perfect confidence? This honors us, and is a blessing to those who put their trust in our judgment, knowlege, purity and honor.—*Medical Standard.*

